# Surveillance of seasonal influenza viruses during the COVID‐19 pandemic in Tokyo, Japan, 2018–2023, a single‐center study

**DOI:** 10.1111/irv.13248

**Published:** 2024-01-05

**Authors:** Hidenori Takahashi, Hiroki Nagamatsu, Yuka Yamada, Naoya Toba, Mio Toyama‐Kousaka, Shinichiro Ota, Miwa Morikawa, Masahiro Shinoda, Syunsuke Takano, Suzuko Fukasawa, Kaeyoung Park, Takahiko Yano, Masamichi Mineshita, Masaharu Shinkai

**Affiliations:** ^1^ Department of Respiratory Medicine Tokyo Shinagawa Hospital Tokyo Japan; ^2^ Department of Infection Control Tokyo Shinagawa Hospital Tokyo Japan; ^3^ Department of Respiratory Medicine St. Marianna University School of Medicine Kawasaki Japan

**Keywords:** COVID‐19 pandemic, immunity, influenza trends, population health, Quick Navi‐Flu2

## Abstract

**Introduction:**

COVID‐19 pandemic led to significant reductions in influenza detection worldwide, fueling debates on whether influenza truly ceased circulating in communities. The number of influenza cases decreased significantly in Japan, raising concerns about the potential risk of decreased immunity to influenza in the population. Our single‐center study aimed to investigate influenza trends before and during the COVID‐19 pandemic in Tokyo, Japan.

**Materials and Methods:**

This cross‐sectional study included patients of all ages who visited Tokyo Shinagawa Hospital between April 1, 2018, and March 31, 2023. Influenza and COVID‐19 tests were conducted using Quick Navi‐Flu2 and polymerase chain reaction (PCR). We analyzed data from before and during the COVID‐19 epidemic, based on patient background, hospitalization, and deaths, collected from medical records.

**Results:**

A total of 12 577 influenza tests were conducted, with approximately 100 tests consistently performed each month even in the influenza off‐season. Throughout the observation period, 962 positive cases were identified. However, no cases were observed for 27 months between March 2020 and November 2022. Influenza A cases were reobserved in December 2022, followed by influenza B cases in March 2023, similar to the influenza incidence reports from Tokyo. The positivity rate during the 2022–2023 winter season was lower than before the COVID‐19 epidemic and decreased in elderly patients, with no hospitalizations or deaths observed.

**Conclusion:**

This single‐center study provided actual trend data for influenza patients before and during COVID‐19 outbreaks in Tokyo, which could offer insights into the potential impact and likelihood of influenza virus infection in Japan.

## 1 INTRODUCTION

Influenza is a respiratory illness that infects 5%–15% of the global population during regular seasonal outbreaks each year. The World Health Organization estimated that these infections cause 3–5 million cases of severe illness and approximately 290,000–650,000 deaths.^1^ However, since the global spread of severe acute respiratory syndrome coronavirus 2 (SARS‐CoV‐2), a significant reduction in influenza detection has been observed in many countries. During the COVID‐19 pandemic, influenza sample collection decreased due to shifting priorities, limited funding, and the closure of outpatient clinics, resulting in a decline in both the number of tests conducted and positivity rates.^2^ Nevertheless, the number of influenza cases reportedly decreased during the 2020–2021 influenza season in countries of the Northern Hemisphere.^3^ In Japan, seasonal influenza epidemics typically begin in early January and peak within a few weeks.^4^ Notably, a prolonged and significant decrease in the number of influenza patients was observed in Japan, with the number of influenza cases reported in winter 2020–2021 estimated as less than 1/1000th of the typical annual amount reported by the National Institute of Infectious Diseases.^5^ The following winter of 2021–2022 saw a new low in the number of influenza cases.^5^ This 2‐year absence of influenza activity in Japan has raised concerns about the potential risk of decreased immunity to influenza, particularly among the elderly population, who are at greater risk of influenza‐related complications and death.^6^, ^7^ The concurrent prevalence of influenza and COVID‐19, often referred to as a “twin‐demic,” has been suggested to lead to a shortage of hospital beds.^8^ In response to the influenza epidemic in the Southern Hemisphere, the potential impact and likelihood of influenza virus infection in Japan during winter 2022–2023 were closely monitored.^4^

With the objectives of preventing nosocomial infections and conducting community surveillance, our institution, a core regional hospital in Tokyo, had implemented a policy of conducting universal influenza antigen testing for all patients with fever on admission before COVID‐19 pandemic. This practice ensured a consistent number of tests was being performed even during the influenza off‐season and against the backdrop of the COVID‐19 pandemic.

This single‐center study appears to represent the only investigation to date that captures the actual trend of influenza patients, conducting assessments of both prevalence and patient demographics while avoiding the impact of reduced influenza testing due to COVID‐19 pandemic.^2^, ^9^

## 2 MATERIALS AND METHODS

### 2.1 Study design

We conducted this investigation as a single‐center cross‐sectional study spanning from April 1, 2018, to March 31, 2023, encompassing periods both before and during the COVID‐19 epidemic. The study was conducted at Tokyo Shinagawa Hospital, a designated major facility for COVID‐19 patient admissions from the 23 wards of Tokyo.

### 2.2 Compliance with ethical guidelines

This study was approved by the ethics committee of Tokyo Shinagawa Hospital (approval no. 22‐A‐14).

### 2.3 Patient recruitment

Patients presenting with fever or upper respiratory symptoms suggestive of influenza underwent diagnostic testing and universal screening for influenza on admission if their body temperature was ≥37.5°C. Approximately 100 influenza tests were performed monthly, regardless of the season. Influenza tests conducted during this period were analyzed retrospectively.

### 2.4 Testing protocol

Patients were tested using the Quick Navi‐Flu2 test kit (Denka Seiken, Niigata, Japan), which offers 94.8% sensitivity and 98.4% specificity for influenza A virus and 97.8% sensitivity and 96.8% specificity for influenza B virus.^10^ Suspected false‐positive results were confirmed using polymerase chain reaction (PCR).

Additionally, the potential for co‐infections with SARS‐CoV‐2 was investigated. The diagnosis of COVID‐19 throughout the observation period aligned with the Japanese “Clinical Management of Patients with COVID‐19” guidelines in effect at the time.^11^

### 2.5 Era definitions

We defined March 2020 as the point at which influenza dynamics changed due to the implementation of travel restrictions and the first emergency declaration, which led to the widespread adoption of nonpharmaceutical interventions against COVID‐19.^12^ The pre‐COVID era was defined as the period from April 2018 to March 2020, while the COVID‐19 pandemic era was defined as the period from April 2020 to March 2023, allowing for a comparison of influenza dynamics between periods.

### 2.6 Data collection

We collected patient information, including age, sex, hospitalization, and deaths, from the medical records.

### 2.7 Statistical analysis

Statistical significance was determined using the chi‐square test for comparing sex, age stratum, hospitalization, and death, and Student's *t* test for comparing average age. We performed all analyses using R version 4.0.3 (R Core Development Team, Vienna, Austria), with the significance level set at *p* < 0.05.

### 2.8 Comparison with broader context

To assess the representativeness of the trends observed for influenza incidence, we compared the findings with reported patient count trends from 419 designated influenza sentinel medical institutions in Tokyo, as documented in the influenza database of the Tokyo Metropolitan Government.^13^

### 2.9 Correlation with COVID‐19 situation

To examine correlations with the prevailing COVID‐19 situation in the city, we conducted a comprehensive analysis by capturing and comparing data from the COVID‐19 database of the Tokyo Metropolitan Government.^14^

## 3 RESULTS

**FIGURE 1:**
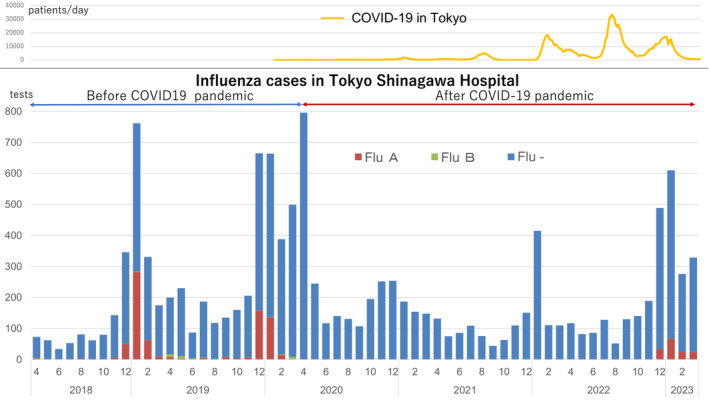
Time series of influenza and COVID‐19 positive numbers by month at Tokyo Shinagawa Hospital between 2018 and 2023. Although an average of 100 influenza tests were performed during the off‐season, no positive cases were observed from April 2020 to November 2022. The number of new COVID‐19 cases in Tokyo has been recorded since February 2020, but fluctuations did not align in parallel with those of influenza.

A total of 12 577 influenza tests were conducted in this study (Table 1), with around 100 tests consistently performed each month even in the influenza off‐season (Figure 1). A total of 962 positive cases were identified (Table 2), but a period of 27 months from March 2020 to November 2022 was identified in which no cases were observed; cases of influenza A were observed again from December 2022, while cases of influenza B were observed again in March 2023. This trend resembled the influenza incidence reports from Tokyo.^5^ Furthermore, the positivity rate during the 2022–2023 winter season (2.2%) was lower than that seen pre‐COVID‐19 (14.1%, *p* < 0.001).

**TABLE 1:** Characteristics of cases tested for influenza at Tokyo Shinagawa Hospital between April 1, 2018, and March 31, 2023 (*N* = 12,577).

	All tested cases (*N* = 12,577)		
	Before‐COVID‐19 (2018/Apr‐2020/Mar)	After‐COVID‐19 (2020/Apr‐2023/Mar)	*p* value
Total patient	5741	6836	
Sex: male (%)	3005 (52.3%)	3836 (56.1%)	<0.001
Age mean ± SD	53.6 ± 26.0	58.0 ± 25.1	<0.001
0–9 (%/test)	222 (3.9%)	1857 (27.2%)	
10–19 (%/test)	165 (2.9%)	138 (2.0%)	
20–29 (%/test)	915 (15.9%)	713 (10.4%)	
30–39 (%/test)	851 (14.8%)	640 (9.4%)	
40–49 (%/test)	610 (10.6%)	445 (6.5%)	
50–59 (%/test)	453 (7.9%)	419 (6.1%)	
60–69 (%/test)	420 (7.3%)	443 (6.5%)	
70–79 (%/test)	783 (13.6%)	754 (11.0%)	
80–89 (%/test)	896 (15.6%)	947 (13.9%)	
90– (%/test)	426 (7.4%)	480 (7.0%)	

*Note*: Total flu testing in children under 10 and seniors over 60 increased pre‐ and post‐COVID‐19, with the overall average age increasing.

**TABLE 2:** Characteristics of patients that tested positive for influenza at Tokyo Shinagawa Hospital between April 1, 2018, and March 31, 2023 (*N* = 962).

	Flu positive patients (*N* = 962)		
	Before‐COVID‐19 (2018/Apr‐2020/Mar)	After‐COVID‐19 (2020/Apr‐2023/Mar)	*p* value
Total patient [test positivity ratio], %	811 [14.1%]	151 [2.2%]	<0.001
Sex: male (patient ratio), %	416 (51.3%)	90 (59.6%)	0.063
Flu A positive (patient ratio), %	765 (94.3%)	150 (99.3%)	0.006
Age mean ± SD, year	41.5 ± 21.6	27.9 ± 17.7	<0.001
0–9 (patient ratio), %	48 (5.9%)	19 (12.6%)	
10–19	39 (4.8%)	26 (17.2%)	
20–29	193 (23.8%)	59 (39.1%)	
30–39	147 (18.1%)	12 (7.9%)	
40–49	130 (16.0%)	17 (11.3%)	
50–59	84 (10.4%)	8 (5.3%)	
60–69	58 (7.2%)	5 (3.3%)	
70–79	56 (6.9%)	3 (2.0%)	
80–89	44 (5.4%)	1 (0.7%)	
90–	12 (1.5%)	1 (0.7%)	
Hospitalization (patient ratio), %	33 (4.1%)	0	0.006
Death (patient ratio), %	2 (0.2%)	0	1.000

*Note*: Flu‐positive patients in children under 10 years old and seniors over 60 years old were decreased before and during COVID‐19, with the overall average age decreasing.

Abbreviation: SD, standard deviation.

In addition, we extracted the background characteristics and clinical courses of patients who underwent influenza testing. The total number of tested cases before and during the COVID‐19 outbreak were 5741 and 6836, respectively. Both the numbers of elderly individuals over 60 years old and children under 10 years old increased, from 2525 to 2624, and from 222 to 1857 respectively. The mean (±standard deviation [SD]) ages of individuals tested during these periods were 53.6 ± 26.0 years and 43.1 ± 24.2 years, respectively.

In contrast, the total number of positive patients decreased from 811 to 151, between before and during the COVID‐19 outbreak. The numbers of elderly individuals and children also decreased, from 170 to 10 and from 48 to 19, respectively. A marked decrease in mean age was recognized, from 41.5 ± 21.6 years before the outbreak to 27.9 ± 17.7 years during the outbreak.

Rates of hospitalization and deaths due to influenza decreased from before to during the COVID‐19 outbreak (hospitalization: 4.1% vs. 0, *p* = 0.012; death: 0.2% vs. 0, *p* = 0.541). Among hospitalized patients, 31 had influenza A and two had influenza B, while both fatal cases were elderly patients in their 60s and 100s with influenza A. In the winter of 2022–2023, all patients were treated on an outpatient basis.

Interestingly, simultaneous testing for influenza and SARS‐CoV‐2 was conducted in 2063 patients, yet co‐infection with both viruses was not observed throughout the observation period.

## 4 DISCUSSION

Our research has revealed a period of 27 months in which community‐acquired influenza infections were absent, from March 2020 to November 2022. During the nonepidemic seasons of influenza in 2018 and 2019, sporadic cases of influenza patients were detected, albeit in small numbers. However, following the COVID‐19 pandemic, influenza appeared to have been temporarily suppressed to near‐eradication levels, irrespective of the season. This phenomenon is supported by epidemiological investigations in Japan, including the measurement of influenza antigens in wastewater, which also pointed toward a significant reduction in the circulation of influenza viruses in the community after the advent of the COVID‐19 pandemic.^15^

The absence of seasonal influenza has been observed to be approximately 1–2 years in various countries in the Northern Hemisphere, but Japan appears to have experienced a slightly longer period.^2^, ^16^ This may be attributed to the sustained presence of known factors such as hand sanitization, universal mask wearing, and reduced international travel in Japan.^17^ Consequently, over the course of this period exceeding 2 years, immunity against influenza among the Japanese population has decreased.^6^ The crude mortality rate of influenza in Japan is 0.1%, with a mortality rate of 2.8% among hospitalized patients; this rate is even higher among elderly individuals 60 years old and above.^18^ Concerns have been raised about an increase in influenza patients, severe cases, and bed shortages, but our data showed that during the 2022–2023 winter season, patient numbers were lower than the winter before COVID‐19, no hospitalizations or deaths due to influenza were observed, nor were any bed shortages due to influenza identified. Although the number of elderly people over 60 years old in all tested cases seemed to have increased somewhat from before to during the COVID‐19 epidemic, the number of positive patients decreased (from 70/2525 patients to 10/2614 patients; *p* < 0.001). The decrease in elderly influenza patients has also been reported from rural areas in Japan, in which the proportion of elderly among the population is high.

For instance, as reported by the rural area of Iwate Prefecture, the proportion of elderly individuals 60 years old and over who had influenza was 7.1% in the 2019–2020 season, decreasing to 2.0% in 2022–2023.^19^

We propose that the absence of a surge in influenza hospitalizations and severe cases, despite a decline in population immunity, can be attributed to a reduction in influenza incidence among the initially high‐risk elderly demographic.

The decline in influenza cases may be attributed to heightened awareness of infectious diseases across the population and the widespread adoption of universal preventive measures, including mask‐wearing,^17^, ^20^ further complemented by a decline in international travel.^21^ Specifically within the elderly population, there was potential for infection prevention benefits arising from lifestyle changes, such as a decrease in outings and an increase in online interactions with family members.^22^ Moreover, efforts were underway to ensure an ample supply of influenza vaccines and to promote vaccination among the elderly in the winter of 2022–2023.^4^, ^23^

A comparison was made between the detection patterns of influenza and fluctuations in new case numbers of COVID‐19 within the Tokyo metropolitan area. The peaks of COVID‐19 and influenza outbreaks do not coincide, and reports from various countries have shown similar trends.^8^ No co‐infection cases with influenza were identified, like reports from the start of the COVID‐19 pandemic,^24^ suggesting that the abovementioned overlapping defense measures made co‐infections less likely.

This study had several limitations. First, viral testing for influenza was performed mainly using a rapid diagnostic test kit, which cannot detect influenza C or other minor influenza viruses. However, no outbreaks of influenza C were reported during the observation period, and a focus on the detection of influenza A and B types was deemed sufficient.^25^ Second, this study was performed at a single institution and involved a relatively small number of patients, particularly regarding children under 10 years old. Third, we did not include information on influenza vaccination or influenza antiviral treatment, as these data could not be consistently obtained for all patients across the various data sources.

This report, as the sole cross‐sectional influenza surveillance conducted in Tokyo, has revealed a distinct period of influenza absence, followed by its resurgence. The study provides evidence that the decrease in the number of influenza tests did not necessarily lead to a decrease in the number of positive cases^9^ and supports the notion that simultaneous infection with influenza and SARS‐CoV‐2 is rare.^24^, ^26^

Additionally, this investigation presents initial evidence confirming a reduction in influenza cases among the elderly during the COVID‐19 pandemic, as alluded to numerically in the database of the Tokyo Metropolitan Government.^13^

We have concerns that a resurgence of influenza might occur after the relaxation of infection control measures such as universal mask wearing and the restoration of active international travel. A follow‐up investigation is therefore required.

## 5 CONCLUSION

This single‐center study conducted influenza surveillance during the COVID‐19 pandemic in Tokyo, Japan, from April 2018 to March 2023. The study showed real‐world data for decreased influenza activity during the pandemic. In fact, a complete absence of influenza cases was apparent at city hospitals in Tokyo for a duration of two and a half years.

## AUTHOR CONTRIBUTIONS

*Conceptualization*: Hidenori Takahashi and Syunsuke Takano. *Methodology*: Hidenori Takahashi, Syunsuke Takano, and Suzuko Fukasawa. *Formal analysis*: Hidenori Takahashi. *Investigation*: Hidenori Takahashi, Syunsuke Takano, and Suzuko Fukasawa. *Resources*: Suzuko Fukasawa. *Data curation*: Hidenori Takahashi. *Writing—original draft preparation*: Hidenori Takahashi. *Writing—review and editing*: Hidenori Takahashi, Hiroki Nagamatsu, Yuka Yamada, Naoya Toba, Mio Toyama‐Kousaka, Shinichiro Ota, Miwa Morikawa, Masahiro Shinoda, Syunsuke Takano, Suzuko Fukasawa, Kaeyoung Park, Takahiko Yano, Masamichi Mineshita, and Masaharu Shinkai. *Visualization*: Hidenori Takahashi, Hiroki Nagamatsu, and Syunsuke Takano. *Supervision*: Masamichi Mineshita. *Project administration*: Masaharu Shinkai. All authors have read and agreed to the published version of the manuscript.

## CONFLICT OF INTEREST STATEMENT

None to declare.

### PEER REVIEW

The peer review history for this article is available at https://www.webofscience.com/api/gateway/wos/peer-review/10.1111/irv.13248.

## Data Availability

Data available on request due to privacy/ethical restrictions.
