# Correction to “Surveillance of seasonal influenza viruses during the COVID‐19 pandemic in Tokyo, Japan, 2018–2023, a single‐center study”Takahashi H, Nagamatsu H, Yamada Y, et al. Influenza and Other Respiratory Viruses. 2024; Volume 18, Issue 1, e13248. DOI: https://doi.org/10.1111/irv.13248

**DOI:** 10.1111/irv.70063

**Published:** 2024-12-25

Takahashi H , Nagamatsu H , Yamada Y , et al., Influenza and Other Respiratory Viruses. 2024; Volume 18, Issue 1, e13248. DOI: 10.1111/irv.13248.38188373 PMC10767599

The originally published article contains an error in the stated duration of the influenza inactivity period. The text incorrectly stated 27 months between March 2020 and November 2022. This was a miscalculation; the correct duration is 32 months. Furthermore, the description of the timeframe (“March 2020 to November 2022”) was imprecise. The actual timeframe spans from late March 2020 to early December 2022. These corrections apply to the following sections:

1. **Abstract**
  • In the Results, the third sentence reads “no cases were observed for 27 months between March 2020 and November 2022.” This should read to “no cases were observed for 32 months, from late March 2020 to early December 2022.”
2. **Results**
  • The second sentence originally reads “but a period of 27 months from March 2020 to November 2022 was identified in which no cases were observed; cases of influenza A were observed again from December 2022, while cases of influenza B were observed again in March 2023.” This should read “but a period of 32 months, from late March 2020 to early December 2022, was identified in which no cases were observed; cases of influenza A were observed again from December 2022, while cases of influenza B were observed again in March 2023.”
3. **Discussion**
  • The first sentence originally reads “Our research has revealed a period of 27 months in which community‐acquired influenza infections were absent, from March 2020 to November 2022.” This should read “Our research has revealed a period of 32 months in which community‐acquired influenza infections were absent, from late March 2020 to early December 2022.”
4. **Figure 1 Legend**
  • In the Figure 1 legend, the third sentence reads “no positive cases were observed from April 2020 to November 2022.” This should read “no positive cases were observed from late March 2020 to early December 2022.”

We apologize for this error.

